# Autophagy cooperates with PDGFRA to support oncogenic growth signaling

**DOI:** 10.1080/15548627.2024.2338572

**Published:** 2024-04-18

**Authors:** Joanne E. Simpson, Noor Gammoh

**Affiliations:** Cancer Research UK Scotland Centre, Institute of Genetics and Cancer, University of Edinburgh, Edinburgh, UK

**Keywords:** Cancer, endocytosis, glioblastoma, PDGFRA, receptor tyrosine kinases, signaling

## Abstract

Macroautophagy (referred to as autophagy hereafter) is a highly conserved catabolic process which sequesters intracellular substrates for lysosomal degradation. Autophagy-related proteins have been shown to be involved in various aspects of tumor development by engaging with multiple cellular substrates. We recently uncovered a novel role for autophagy in regulating the signaling and levels of PDGFRA, a receptor tyrosine kinase amplified in several cancers. We discovered that PDGFRA can be targeted to autophagic degradation by binding the autophagy cargo receptor SQSTM1. Surprisingly, PDGFRA-mediated signaling is perturbed in the absence of autophagy despite enhanced receptor levels. We show that this is due to disrupted trafficking of the receptor to late endosomes where signaling activity persists. Conversely, prolonged autophagy inhibition results in a transcriptional downregulation of *Pdgfra* as a result of inhibited signaling activity demonstrating that short- and long-term autophagy inhibition have opposing effects on receptor levels. We further investigated the consequence of PDGFRA regulation by autophagy using a mouse model for gliomagenesis where we observed a disruption in PDGFA-driven tumor formation when autophagy is inhibited. Activation of downstream signaling through *Pten* mutation overrides the need for autophagy during tumor development suggesting a genotype-specific role for autophagy during tumorigenesis. Altogether, our findings provide a novel mechanism through which autophagy can support tumor growth.

## Main text

Autophagy has been associated with a wide range of diseases. This is a result of a plethora of cellular substrates that can be targeted to degradation via autophagy. In cancer, inhibiting autophagy has been sought as a promising approach to enhance response to treatment. However, the role of autophagy in cancer needs to be carefully studied to dissect the dependency on the mutational burden and stage of tumor progression.

**Figure 1.**
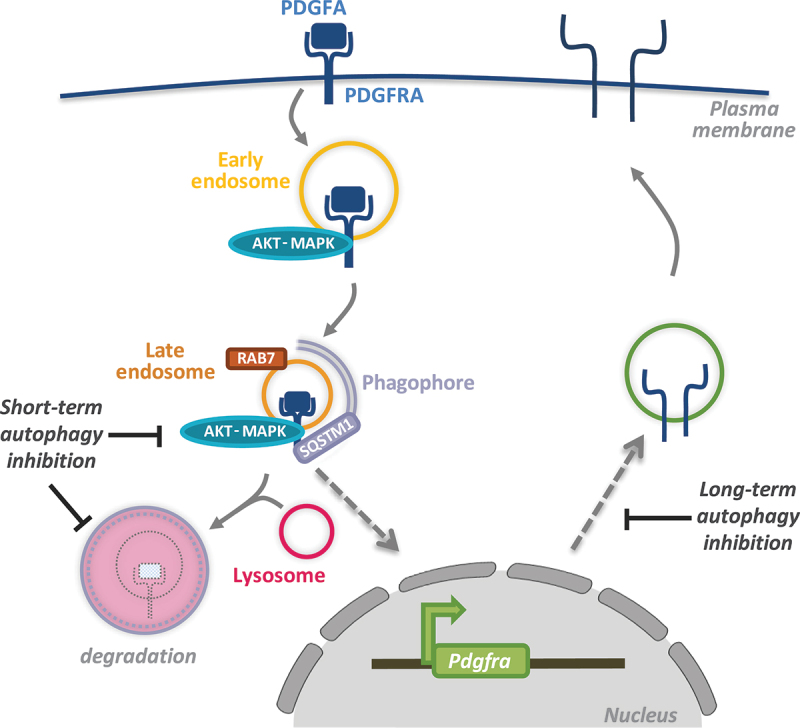
A summary of the various aspects of PDGFRA regulation by autophagy. Following receptor endocytosis upon binding to its ligand PDGFA, PDGFRA is trafficked to late endosomes marked by RAB7 where signaling activity can persist. In the absence of autophagy, receptor trafficking to late endosomes is disrupted leading to reduced signaling despite enhanced PDGFRA levels due to inhibited lysosomal degradation. In the long term, disrupted PDGFRA signaling in autophagy-inhibited cells leads to a transcriptional downregulation of *PDGFRA* resulting in reduced cellular availability of this receptor.

Whereas multiple studies have investigated how autophagy can influence the growth of established tumors and, more recently, in metastasis, less is known about how autophagy plays a role during tumor initiation. Our recent findings show that autophagy can regulate pro-growth signaling during the activation of PDGFRA (platelet derived growth factor alpha) [1]. PDGFRA is a receptor tyrosine kinase (RTK) and is one of the commonly mutated oncogenes that drive tumor growth in glioblastoma, the most aggressive and lethal brain tumor. We show that PDGFRA, when activated upon binding its ligand PDGFA, can be targeted to phagophores for degradation by binding the cargo receptor SQSTM1/p62 (Figure 1). Acute inhibition of autophagy enhances PDGFRA levels in cells, but this is associated with reduced receptor activity as a result of its perturbed trafficking to late endosomes. This finding suggests that autophagy plays a dual role during PDGFRA signaling. On the one hand, autophagy supports PDGFRA signaling by facilitating its recruitment to late endosomes where signaling can be sustained for this RTK. On the other hand, the targeting of PDGFRA to lysosomes is enhanced by autophagy and results in signal termination. This dual regulation of PDFFRA by autophagy may be advantageous to prevent unwanted hyperactivity of this receptor in non-transformed cells.

Further mechanistic studies are required to fully understand how autophagy can target PDGFRA for degradation. As SQSTM1 binds ubiquitinated targets, it would be interesting to determine whether ubiquitination is required for receptor recognition and the ubiquitin ligase involved. Furthermore, how autophagy can target the degradation of membranous proteins requires further analysis; whether this involves piecemeal degradation or phase separation remains to be determined. In addition, since RTKs are targeted for lysosomal degradation upon their activation and endocytic trafficking, it remains to be determined why cells employ autophagy to facilitate the lysosomal degradation of PDGFRA and indeed other receptors previously shown to be targeted by autophagy, such as MET/c-Met.

While the above findings were derived from examining cells after acute autophagy inhibition (up to 16 h post siRNA transfection to target autophagy-related gene products or small molecule inhibitors targeting ULK1), we were surprised to observe a reduction in PDGFRA protein levels upon prolonged autophagy inhibition, for example after CRISPR-Cas9-mediated gene disruption targeting key autophagy players. Further analysis showed that, in the absence of autophagy, *Pdgfra*, but not related RTKs including *Pdgfrb*, is transcriptionally downregulated (Figure 1). This is likely to be a mechanism of adaptation in the absence of autophagy and concurrent reduction in receptor activity which, in a positive feedback loop, can regulate *Pdgfra* expression. Interestingly, reduced phosphorylation and thus activation of the transcriptional factor CREB, previously shown to facilitate *Pdgfra* transcription, is observed in cells with inhibited autophagy. Whether other CREB transcriptional targets are also affected in the absence of autophagy remains to be further investigated.

This post- and pre-transcriptional regulation of PDGFRA by autophagy may have a number of consequences. Our study shows that in the absence of autophagy, PDGFRA-driven brain tumor formation is inhibited. This is likely to be a result of reduced receptor signaling in cells lacking autophagy as tumorigenesis can be restored by activating AKT/PKB signaling upon *pten* deletion. While this may have important implications during anti-cancer treatment, further studies are still required to demonstrate whether autophagy inhibition can be an effective means to reduce tumor burden by inhibiting autophagy after tumor formation. The clinical implications of such findings may be complicated by tumor heterogeneity and the complex mutational landscape of cancers cells, especially in the case of aggressive cancers such as glioblastoma. Nevertheless, identifying PDGFRA as a novel autophagy substrate is likely to shed valuable insights when considering autophagy as a target in disease.
